# Open-mindedness and phenomenological psychopathology: an intellectual virtue account of phenomenology and three educational recommendations

**DOI:** 10.1080/09515089.2024.2379987

**Published:** 2024-07-22

**Authors:** Andrew Jonathan Maile

**Affiliations:** Institute for Mental Health, University of Birmingham, Birmingham, UK

**Keywords:** Intellectual virtue, open-mindedness, phenomenological, psychopathology, education

## Abstract

In his account of phenomenological psychopathology, Karl Jaspers advocates for the central role of subjective experience, something which he maintains cannot be accessed through intellectual effort, but through “empathic understanding” alone. In contradistinction to Jaspers’ account, I propose that phenomenology, as a process of inquiry and investigation, is fundamentally epistemological. Accordingly, I offer an intellectual virtue characterization of phenomenological psychopathology, using open-mindedness to illustrate the close conceptual links between the phenomenological endeavor and the intellectual virtues. By introducing the intellectual virtue lexicon into the phenomenological psychopathology discourse, I then offer three preliminary recommendations for the training and education of phenomenological clinicians. Centering the educational recommendations on the psychiatric interview, I suggest that good questioning, listening, and reflecting necessarily require cultivation for intellectually virtuous phenomenological inquiry.

## Introduction

“Phenomenological Psychopathology” has garnered much recent interest, alongside related aspects in the philosophy of psychiatry, seeking to establish the epistemic rights of the psychiatric patient as the knower and expert of their lived experiences (see Feyaerts et al., 2021; Ritunnano et al., 2022a, 2022b, for a phenomenological approach to delusions). This renewed interest is often offered in response to a dominant biomedical model of psychiatry that risks alienating individuals with psychosis from professionals (and associated institutions) whose occupational purpose is to support them through the vicissitudes of their experiences. In support of the reinvigoration and “renewal” of phenomenological psychopathology, this paper seeks to advocate for the introduction of the intellectual virtue lexicon into the phenomenology discourse. By offering such an account, I aim to highlight the complementarity between the intellectual virtues and phenomenological psychopathology.

This account is offered in contrast to Jaspers’ claim that access to the inner life world of lived experience cannot occur through intellectual effort, but rather through “empathic understanding” (1968). By positioning phenomenological psychopathology as an act of inquiry and investigation, I propose that it is fundamentally epistemological, seeking truth, knowledge, and understanding of the lived psychic experience. Following this claim, I offer an intellectual virtue account of phenomenology, and use open-mindedness as an exemplar virtue to illustrate the valuable contribution of incorporating the intellectual virtues into phenomenology discourse. Finally, I make three educational recommendations for intellectually virtuous phenomenological clinicians. By using the psychiatric interview as a springboard, I suggest that the intellectual skills of good questioning, listening, and reflecting, need to be intentionally cultivated in clinician education and training.

My hope is that, by including the intellectual virtue lexicon into the phenomenological psychopathology discourse, this will support efforts to reconstruct phenomenological psychopathology in a way befitting its position as an interdisciplinary and democratic discipline in the 21st century.

## Phenomenological investigation and lived experience

At its heart, phenomenological psychopathology is concerned with investigating the lived experience of individuals whose psychology is considered to operate contrary to the way “normal” has been defined within our society.^1^ It is interested in psychic phenomena, and the subjective or lived dimensions of such phenomena. This focus on lived experience relies upon a distinction between objective and observable symptoms, and those that are subjective – known only by the psychiatric patient. This focus on the subjective is what sets phenomenological psychopathology apart from more dominant forms of biomedical psychotherapeutic processes. Karl Jaspers, whose revolutionary work established the phenomenological approach in psychopathology, identified that the subjective symptoms of the psychiatric patient belong to their elusive inner life, the assessment of which is challenging (Jaspers, 1968).

Phenomenology “begins with the premise that the analysis of direct description can provide significant understanding and insight into the nature of the experience itself” (Upthegrove et al., 2016, p. 88). Thus, the phenomenological method in psychopathology is a process of investigation and analysis that seeks to ascertain and elucidate the subjective experience of psychopathology, to lay bare the “essence” of individual’s lived experiences (Broome et al., 2013). Such a commitment is independent from considerations of pathological causation, labeling of clinical features, or the observation of signs and symptoms typical of pathologizing clinical conditions. Rather, the focus is to gain an understanding of the experience from a first-person perspective, following a process of systematic subjective investigation:

… phenomenological psychopathology can be conceived of as *psychopathologia prima*. It assumes that the primary object of psychiatry is the patient’s subjectivity, thus putting all its efforts into focussing on the patient’s state of mind as it is experienced and narrated by them. (Stanghellini & Broome, 2014, p. 170)

Indeed, because phenomenological psychopathology centers on gaining an “understanding of the ‘world’ a patient lives in” (Stanghellini & Broome, 2014, p. 170) through a process of subjective investigation, this undertaking necessarily rests on epistemological grounds. It is this epistemological foundation of phenomenological psychopathology which I seek to explicate in Section II by offering an intellectual virtue account of it.

### Phenomenological understanding and empathy

Jaspers suggests moving beyond merely identifying the content of the psychopathological condition, and that for the lived experience of the patient to be adequately grasped, this requires empathic understanding. For Jaspers, to understand the psychiatric patient’s subjective phenomena requires “transferring oneself, so to say, into the other individual’s psyche” (Jaspers, 1968, p. 1313), the achievement of which relies upon empathy. This “empathic understanding” enables access to the patient’s lived world experience. Jaspers distinguishes between two modes of understanding, rational and empathic:

Rational understanding always leads to a statement that the psychic content was simply a rational connection, understandable without the help of any psychology. Empathic understanding, on the other hand, always leads directly into the psychic connection itself. Rational understanding is merely an aid to psychology, empathic understanding brings us to psychology itself. (Jaspers, 1997, p. 304)

While the focus on empathy, or “empathic”, is central to Jaspers' account of understanding, such that it enables access to the patient’s life world, my particular aim here is to focus on *understanding*, and not exclusively it’s “empathic” nature or mode. For Jaspers, the focus on empathy was crucial to understanding, because he maintained that it was only through empathy that the subjective symptoms of the psychic event could be accessed: “they can only become an inner reality for the observer by his participating in the other person’s experiences” (Jaspers, 1968, p. 1313). Accordingly, accessing another person’s experiences can occur through empathy alone, “not by any intellectual effort” (Jaspers, 1968, p. 1313). Yet, as I hope to clarify in this paper, the very attempt or ability to access another’s life world, contrary to Jaspers’ claim, relies heavily on intellectual effort. In particular, I suggest that the intellectual virtues are foundational to such an ability (see Section II).

Evading the perception of sense-organs and inaccessible through “intellectual effort”, Jaspers classified subjective symptoms according to three categorizations. The first relates to one’s “emotions” and “inner processes”: feelings such as joy, sorrow, or fear. The second relates to psychic phenomena and experiences; descriptions provided by patients which become accessible to the clinician “secondhand through the patient’s own judgment and presentation” (Jaspers, 1968, p. 1313). The third subjective symptom involves “mental processes”, which can be manifested in the patient’s actions and the way they conduct their life. Written at a time when the dominant form of psychology focussed on objective symptoms, “objective psychology” was seen to be in opposition to an interest in subjective symptoms – “subjective psychology”.^2^ Accordingly, Jaspers identified that a “very definite contrast of values” (Jaspers, 1968, p. 1313) existed between the two. To this end, subjective symptoms were taken to be “unreliable for making final judgements and unfruitful for the purpose of any further scientific investigation” (Jaspers, 1968, p. 1313).

This contrast of values, and distinction between “reliable scientific investigation” and the subjective, “elusive inner life”, also appears to have influenced Jaspers’ theory. Accordingly, the former – being scientific – requires cognition and “intellectual effort”, while the latter – which is not scientific – is intellectually inaccessible (and cannot be derived from cognitive processes). Rather, it requires a deeper, psychological engagement through “empathic understanding”. Yet the term “empathy” – which has captured the interest of philosophers since the term’s introduction into our vocabulary in 1873– “continues to defy easy definition” (Spencer & Broome, 2023, p. 2). Indeed, the definition of the term greatly depends on the context and intention of its use. In phenomenology, it is understood to be a means of intentionally experiencing the other (Gallagher & Zahavi, 2012; Spencer & Broome, 2023; Zahavi, 2014).

Theodore Lipps considered knowledge to comprise of three distinct domains, one of which was “knowledge of others”, the distinct cognitive source for which is empathy (Zahavi, 2014, p. 130). For Edmund Husserl (1962), empathy enabled one to intentionally access a “foreign ego”. Similarly, Edith Stein uses the term “empathy” to capture the experience of foreign consciousness. For Stein, empathy “is the basic cognitive source for our comprehension of foreign subjects and their experiences” and involves “complex kinds of social cognition” (Stein, 2010; Zahavi, 2014, pp. 133–134). While it is not clear that Husserl’s account of empathy involves intellectual effort and cognitive processing, the accounts offered by Lipps and Stein arguably do.^3^ Accordingly, this offers “an indirect comprehension of the other that is derivative and refers back to empathy understood as a more basic experiential grasp of the other’s experience” (Zahavi, 2014, pp. 134–135). Stein (1989) was particularly concerned with *what* empathic experience consisted of. For Stein, empathic experiences – or empathic understanding related to perception of experience – was something “generated by cognitive process” (Ratcliffe, 2012, p. 476). While not wading into the debate concerning the vast and murky water of defining empathy, I reference compelling accounts of empathy from other phenomenologists to illustrate the importance of intellectual effort and cognitive processing that is missing from Jaspers’ account.

This characterization of empathy differs from the “mystical or even magical process” (Sass, 2013, p. 98) that enables the psychiatrist to “transform oneself … into the other individual’s psyche … by participating in [their] experience” (Jaspers, 1968, p. 1315). Rather, it suggests that such a process is both complex and cognitively demanding, and arguably still enables the clinician to “gain an essentially personal, indefinable and direct understanding” of the patient’s inner life (Jaspers, 1968, p. 1315).

### The epistemic goals of phenomenology

Reflecting on the role of “understanding” in psychiatric healthcare, Spencer and Broome (2023) advise that the questions asked in the psychiatric interview initiate “an excavation into the internal and ineffable mental life of the psychiatric patient” (p. 4). While objective symptoms may be more readily discernible through observation, accessing the patient’s lived experience, or “life-world”, is not. It is thus that “understanding” (“Verstehen”) or “perception of meaning”, features so centrally in Jaspers’ work (Jaspers, 1997, p. 27). Jaspers distinguishes between “understanding” (Verstehen) – which is “the understanding of psychic events ‘from within’” – and “explanation” (Erklären), “the appreciation of objective causal connections … seen ‘from without’” (Jaspers, 1997, p. 28). His interest, in this sense, is accessing that which exists within, namely something that would remain inaccessible if it were not for the relational and dialectical exchange between patient *qua* knower and clinician *qua* seeker.^4^

According to Hans-George Gadamer, dialogue is central to the medical encounter. For Gadamer, the “art of healing” relies upon “the communicative flow of the patient’s life experience”, yet it also requires moving beyond the patient merely offering their testimony for the psychiatrist or doctor to receive and affirm (Gadamer, 1996, p. 138). Rather, an exchange is required, a process of exploration and interpretation, which occurs dialogically as the patient shares the various meanings that saturate their psychic experiences. This dialogical endeavor includes subjecting the meanings provided by the patient to different processes that can enrich the understanding of those meanings. In this way, the rich and dynamic experiences of the psychic phenomena should be matched by the energy and complexity with which the dialogical interpretation takes place in practice (Gadamer, 1996; Spencer & Kidd, 2023). This dialogical process takes time – it is an “ongoing process” – and commitment – in the form of a “relationship” – that can include both “disorientation” and a “regaining [of] equilibrium” (Gadamer, 1996, p. 137) that ultimately results in the “merging of horizons” and the creation of shared understanding of the psychic phenomena (Gadamer, 1996, p. 112).

Psychopathological conditions can often, indeed typically, be experienced in profoundly unusual ways.^5^ Moreover, the idiosyncratic nature of these experiences defies universal translation or straightforward description, despite attempts to do so from the biomedical scientific approach, which seeks to pathologize according to empirically observed “objective” symptoms. Rather, it is the subjectivity of psychic events and experiences that is key to phenomenological psychopathology. As such, the healthcare professional is called upon to be “the investigator of meaning” (Jaspers, 1997, p. 314), navigating vast and varied interpretations, and seeking to understand the psychic experience. The psychopathology endeavor is to serve “as a bridge” by “providing the basic tools to make sense of mental suffering”, and to pursue “the discovery of new psychopathological knowledge” (Stanghellini & Broome, 2014, p. 170). Indeed, for Jaspers, the hopeful outcome of phenomenological psychopathology is to “further *enrich our knowledge* of what the psychiatric patient really experiences” (Jaspers, 1968, p. 1323, emphasis mine).

The discovery of new knowledge, the investigative pursuit of understanding,^6^ and the establishment of meaning-making to better grasp the elusive inner life – or internal, subjective truth,^7^ of the psychic experience: these are three goals of phenomenological psychopathology. Framed differently, phenomenological psychopathology involves investigating the subjective life-world of the psychiatric patient in the pursuit of understanding and meaning-making. By engaging in a process of meaning-making, the healthcare practitioner is able to grasp the truth of the patient’s subjective, lived experience. These processes ultimately lead to the discovery and generation of new psychopathological knowledge. Truth, knowledge, and understanding: these are also typically considered to be epistemic goals.

## A virtue epistemology account of phenomenology

Acknowledging the epistemic goals of phenomenological psychopathology complete, it thus enables us to consider how one might facilitate success in the phenomenological pursuit of truth, knowledge, and understanding. In this respect I draw upon the work of two influential virtue epistemologists, Linda Zagzebski (1996) and Jason Baehr (2011a), whose work has significantly advanced this growing field of research, and whose position on virtue responsibilism represents the intellectual virtue account I propose in this paper. For Zagzebski (1996), knowledge equates to true belief which is generated, or arises, from “acts of intellectual virtue” (p. 271). Indeed, the intellectual virtues present a compelling case for phenomenological psychopathology.

A virtue, briefly, is a human excellence, and pertains to the possession of a quality that makes one an excellent person (Battaly, 2015; Russell, 2013).^8^ Accordingly, virtue is a lasting, personal feature: “a tendency for the person to be a certain way” (Annas, 2011, p. 8).^9^ Virtues are considered *intellectual* when they contribute to one’s “personal intellectual worth”, enable the production of intellectual goods, and describe how one is as a thinker (Baehr, 2011a, Battaly, 2015, p. 92; Kotzee et al., 2021). Intellectual virtues are thus excellences that enable the production of truth (or true beliefs) and knowledge (Battaly, 2015). In the context of inquiry, which involves “an active and intentional search for the truth about some question”, the intellectual virtues play an important role (Baehr, 2011a, p. 18).

In their pursuit of subjective understanding, the clinician (*qua* seeker) is exercising cognitive agency. A cognitive agent is one who invests their cognitive capacities, experiences, skills, and training – perhaps even their “self-trust” or professional, expert discretion^10^—in the pursuit and attainment of truth (Zagzebski, 2012). Such pursuit of truth places significant demands on both cognitive attitude and agency, requiring the exercise of traits, including intellectual carefulness and thoroughness (Baehr, 2011a, 2016). Accordingly, Baehr (2011a) suggests the demands that inquiry places upon the cognitive agent requires the possession and enactment of intellectual virtues, equipping the agent to either meet or overcome these demands.

Importantly, not all knowledge is necessarily acquired by pursuing the truth about some question, nor is an active or intentional search always necessary. Baehr suggests that this includes most memorial and introspective knowledge (e.g., that I ate a sandwich for lunch, or that my back is sore), identifying the appearance of one’s surroundings, and some a priori knowledge (e.g., some simple arithmetic). Such knowledge “is relatively immediate and automatic; it requires little more than the brute or default operation of our basic cognitive faculties” (Baehr, 2011a, p. 18).^11^ This type of knowledge, however, is not the type of knowledge after which phenomenological psychopathology is seeking. Indeed, subjective knowledge of lived experience and psychic phenomena is neither immediate nor automatic.

On the contrary, the knowledge after which phenomenological psychopathology seeks is difficult to access. It requires engagement and dialogical exchange with lived experience individual’s, who, through willing engagement in the phenomenological process, make subjective knowledge available through their own “judgement and presentation” (Jaspers, 1968, p. 1313). As Baehr (2011a) argues, such knowledge is typically accessed through the act or process of inquiry. It places cognitive demands upon the agent, requiring thought, judgment, reasoning, evaluation, adjudication, interpretation, and reflection. Acquiring this information requires effort, and barriers to its access exist. This, I suggest, is quite the same for the subjective knowledge after which the phenomenological psychopathology method seeks to access.

Arguably, acquisition of the subjective information (truth, knowledge, and understanding) which phenomenological psychopathology is seeking is especially “difficult to come by”. For it relies upon a therapeutic relationship, where one cognitive, epistemic agent (the clinician *qua* seeker) is requiring access to information, information which is accessible only through another cognitive, epistemic agent (the patient *qua* knower). In this context of inquiry, the clinician *qua* seeker– ’the investigator of meaning’ – is not merely grappling with affairs of ancient history, and accessing “difficult to come by” texts in ancient languages, or microscopic cell processes of a biological nature. Nor indeed does the inquiry rely upon objective symptoms, combining perception by the senses and theoretical analysis of their “derivation”, the measurement of performance, nor the analysis of biomedical features.^12^

Rather, the phenomenological psychopathologist (clinician *qua* seeker) is required to access and navigate vast and varied interpretations and representations of psychic phenomena, and then to investigate and analyze the subjective and elusive inner life of the patient *qua* knower.^13^ Such inquiry relies upon the successful navigation of a dynamic relationship, and upon the possibility of testimonial exchange (Fricker, 2007). This requires the effort and intention of two people working together to achieve what may be no more than a “vague and unconscious understanding”, relying upon “the special attitudes and aptitudes of particular individuals” (Jaspers, 1968, p. 1315).^14^ Moreover, as Jaspers (1968) identified, due to the typical complexity of psychic phenomena, these experiences are not easily articulated. The clinician is called to be “a bridge between the human and clinical sciences, providing the basic tools to make sense of mental suffering … [to scrutinize] the diverse and varied nature of what is really there in the patient’s experience … [and to enable] the discovery of new psychopathological knowledge” (Stanghellini & Broome, 2014, p. 170).^15^ Indeed, Jaspers conceptualized “understanding” to be a form of “knowledge that can be communicated, investigated and argued about” (Jaspers, 1968, p. 1315). To this end, it is difficult to justify a phenomenological psychopathology that not does not rely extensively upon “intellectual effort” and intentional, effortful inquiry.

Such a demand of intentional, effortful inquiry by the clinician *qua* seeker through the method of phenomenological psychopathology is thus also the domain of personal, and especially intellectual, character. As Baehr (2011a) argues:

An intellectually virtuous person is one who thinks, reasons, judges, interprets, evaluates, and so on, in an intellectually appropriate or rational way … Thus where cognitive success requires inquiry, it also typically requires an exercise of one or more intellectual character virtues. (p. 18–19)

It thus seems highly plausible that, where the phenomenological endeavor requires success in the realm of thinking, reasoning, judging, interpreting, evaluating, and so on – processes fundamental to inquiry and equally fundamental to accessing the subjective inner life world – this will also require – or at minimum be benefited by – exercising one or more of the intellectual virtues.

### Ensuring excellence in phenomenological inquiry

Baehr (2011a) suggests that, for an inquiry to be successful, certain demands are placed upon it: motivation, focus, and accessing and evaluating various sources. The first demand is motivational – while an intellectually lazy or ignorant inquirer is unlikely to initiate an inquiry that requires intention and effort, one who is motivated to pursue truth, knowledge, and understanding, will. Indeed, intellectual virtues like curiosity, contemplativeness, wonder, and reflectiveness, are essential to the success of such a pursuit. In the case of phenomenological psychopathology, the curious clinician will be inclined to be an “investigator of meaning” with respect to individual’s significant, subjective experiences. She will also be motivated to ask questions that enable complex personal experiences to be understood – that enable her to enter the patient’s life world – which will feed her desire for inquiry. Similarly, the reflective, contemplative clinician will be inclined to reflect on what the patient (*qua* knower) shares with her, further motivating her inquiry and informing her questioning.

Motives, as Philippa Foot (1978) and Zagzebski (1996) suggest, which nourish or inform our motivations, are important to an understanding of virtue: they are typically forms of emotion and are action guiding. This connection between the virtuous acts of the clinician, and the clinician’s emotions or feelings, is important. Zagzebski argues that a motive “includes something about *why* a state of affairs is desired”, and “includes something about the way my emotions are tied to my aim” (1996, p. 129). For the clinician, *why* is it important to understand the patient’s lived, subjective experience? The likely answer is because the patient is the expert, the only avenue for subjective, psychic-related information to be accessed.

Empathy, we know, is central to Jaspers’ conceptualization of phenomenological psychopathology, hence the centrality of “empathic understanding” in his account. I suggest that this empathic understanding – the desire to empathize with the patient – serves as the motive for the application of the intellectual virtues in the pursuit of truth, knowledge, and understanding when it comes to the patient *qua* knower’s lived experience. The motive for Jaspers in this regard is to “understand other people, not through considering and analysing their mental life” – in the form of analyzing objective, observable symptoms – “but by living with them in the context of events, actions and personal destinies” (1968, p. 1315). As such, empathy, understood as an emotion or feeling (the motive) initiates and directs the actions of the clinician toward virtuous inquiry (the end).

The second demand is to remain focussed (Baehr, 2011a). For phenomenology, the context of inquiry involves rich, dynamic, and varied psychic phenomena. This necessitates that the dialogical interpretation should match the energy and complexity in which it takes place, and with attention to the fine-grained features of the subjective account (Baehr, 2011a; Gadamer, 1996; Spencer & Kidd, 2023). This demand may require of the clinician certain intellectual virtues, such as perceptiveness, scrutiny, attentiveness, or sensitivity to detail. The scrutinizing clinician will, for example, adopt an appropriately critical mind-set to the subjective content shared by the lived experience expert. Similarly, the perceptive clinician *qua* inquirer will ensure homing in on salient issues and details from the testimonial exchange (Baehr, 2011a; Fricker, 2007).

The third demand calls for the inquirer to consult with and evaluate a wide variety of sources, and to discern which to accept or reject (Baehr, 2011a). This demand concerns the criteria used to evaluate different views, and the temptation to evaluate certain standards or views more favorably – by utilizing relatively lax criteria – than others. Similarly, when views are evaluated over a long period of time, the standards applied to their evaluation may differ. Accordingly, for the clinician to engage in successful inquiry this may require the virtue of intellectual fairness or intellectual humility, ensuring the same evaluative criteria are applied equally across time and case.

A strong, and complex example of such a view might lead to epistemic testimonial injustice,^16^ specifically the *prejudice condition* proposed by Byskov (2021). Such an injustice occurs when prejudice results in the unfair discrimination of someone as a knower (Fricker, 2007, 2017). In the case of phenomenological psychopathology, the intellectually humble or intellectually fair clinician would ensure that the same evaluative criteria are afforded to all the patient *qua* knower’s they engage with, regardless of personal characteristics, such as “gender, social background, ethnicity, race, sexuality, tone of voice, accent, and so on” (Byskov, 2021, p. 121). Intellectual virtues such as intellectual humility or intellectual fairness would also prevent the clinician from granting excess credibility – using more lax evaluative criteria – to a patient *qua* knower when they present with a particular accent, or social and cultural background.^17^

The fourth demand requires the clinician to “confront and process new evidence”, to be aware of the evidence the clinician already has, and “how it bears on the propositions we accept or are considering” (Baehr, 2011a, p. 20). This calls for the intellectual virtues of self-scrutiny and self-awareness. Furthermore, this might require of a clinician “abandoning a belief, suspending a judgement, or conducting further inquiry” (Baehr, 2011a, p. 20). For a clinician to succeed at this, open-mindedness and intellectual humility will likely support the success of such an endeavor.^18^

Baehr (2011a) proposes two additional challenges to the act of inquiry which he deems to be less common. These occur when the subject matter under investigation is “extremely complex and demanding”, or investigating atypical or unusual ways of thinking that requires “an unusual amount of exertion or endurance” (Baehr, 2011a, pp. 20–21). These two challenges seem inherent (although not exclusively necessary) to phenomenological psychopathology. In the context of phenomenology, the clinician is required to set aside “theories and psychological constructs” and turn to that which the clinician “can understand as having real existence” in the patient *qua* knower’s experience (Jaspers, 1968, p. 1316). This calls for the clinician to “think outside the box”, requiring virtues like open-mindedness, intellectual flexibility, and imaginativeness (Baehr, 2011a). Furthermore, this process “is itself a very difficult task … that is laboriously acquired after prolonged critical work and much effort” (Jaspers, 1968, p. 1316). Depending on the particularities of the context of inquiry, this may call upon the clinician for intellectual virtues such as patience, courage, or tenacity.

In this section, leaning heavily on Baehr’s (2011a) work, I hope to have offered an account for the intellectual virtue’s centrality and inherent contribution to successful inquiry in the phenomenological endeavor. The intellectual virtues are fundamentally interconnected, and many apply to an intellectual virtue account of phenomenology (see Maile, forthcoming). In the following section I use open-mindedness to illustrate the value of incorporating an intellectual virtue lexicon into the phenomenology discourse.

### Open-mindedness

The ideal of open-mindedness has its origin in Greek philosophy, most notably captured by the method of Socratic dialogue, which requires interlocutors “to follow the argument where it leads” (Hare, 2011, p. 9, citing Hare, 2009a, p. 7). Open-mindedness is thus concerned with overcoming any obstacles that might impede the serious examination of evidence. That open-mindedness is an intellectual virtue is little contended; William Hare (2009b) argues that it has been recognized as one throughout philosophy's history, and Wayne Riggs (2010) notes it is commonly present at the top of any list of epistemic virtues.

Open-mindedness is often conflated with the concept of “openness” as a personality trait. Openness, one of the five dimensions of personality, is distinct from open-mindedness in several ways. First, it is considered something that is heritable, and stable throughout adulthood – thus, unlike virtue, it cannot be cultivated. Second, it relates to the permeability of consciousness, desirous of new ideas and experiences. Accordingly, it is not morally motivated, nor is its enactment epistemically goal directed (McCrae & Greenberg, 2014). Assuming a neo-Aristotelian virtue ethical understanding of virtue (as I do in this paper), it is more appropriate to think of intellectual virtues in terms of character instead of personality. Indeed, character (which subsumes an agents “inner world”, motivations, and emotions) is typically understood as a subset of personality (Kristjánsson, 2015). Accordingly, virtues such as open-mindedness are morally evaluable, reason-responsive, and educable. Personality traits, in contrast, “are to be understood *amorally* as broad profile dispositions … unchanneled to specific situations” (Kristjánsson, 2015, p. 46). Openness may also be conceptualized as an attitude that enables a degree of hermeneutical flexibility.^19^ Ritunnano (2022) suggests that, with hermeneutical flexibility enabled by an attitude of openness, the interlocutor would be receptive to, and accepting of, co-existing and competing views. This “attitude of openness” is, I suggest, captured by the epistemic virtue of open-mindedness.

Jaspers (1968) argued that phenomenological psychopathology requires the ability to set aside previously held theories, constructs, and “materialist mythologies of cerebral processes”. This requirement aligns well with Baehr’s (2011b) account of open-mindedness,^20^ which I draw upon here to illustrate my case:

An open-minded person is characteristically (a) willing (and within limits) able (b) to transcend a default cognitive standpoint (c) in order to take up or take seriously the merits of (d) a distinct cognitive standpoint. (Baehr, 2011b, p. 202)

In Section I above I have explicated that the goal of the clinician *qua* seeker in the context of phenomenological psychopathology is to gain truth, knowledge, and understanding regarding the patient’s subjective experiences of psychic phenomena. To achieve this goal, a certain openness is required of the clinician, whose main object of inquiry is human subjectivity.^21^ Indeed, according to Stanghellini and Broome (2014), “phenomenological psychopathology is ‘open’ to an unusual extent” (p. 170). Such “openness” is required of the clinician because the phenomenological approach “reveals aspects of experience that other approaches tend to overwrite or eclipse with their strong theoretical claims” (Stanghellini & Broome, 2014, p. 170). Thus, the clinician *qua* phenomenologist needs to allow themselves “freedom from preconception” and to “set aside” previously held theoretical knowledge (and any preference for relying upon it; Jaspers, 1968). These requirements for phenomenological psychopathology, as outlined by Jaspers (1968), and Stanghellini and Broome (2014), reveal a close conceptual link between open-mindedness and phenomenological psychopathology, especially condition (a) and (b).

Open-mindedness calls for an attempt to comprehend or understand “new or challenging subject matter”, or the need to “imagine or conceive of possibilities and alternatives” (Watson, 2022, p. 3). For Baehr, a salient feature, and “conceptual core”, of open-mindedness is the necessary transcendence of preconceptions. Baehr (2011b) argues that an open-minded person necessarily “*departs* or *detaches* from … *moves beyond* or *transcends*, a certain default or privileged cognitive standpoint” (p. 199, italics original). This salient feature of open-mindedness mirrors Jaspers’ concern over “theoretical prejudice” (Jaspers, 1997). As Baehr calls for the transcendence of a “default” and “privileged cognitive standpoint”, Jaspers calls for a suspension of “basic constructs or frames of reference” along with “all outmoded theories, psychological constructs or materialist mythologies of cerebral processes” (1968, p. 1316). In their commentary on Jaspers’ work, Spencer and Kidd (2023) maintain that “this bracketing includes the taxonomy and classification pre-established in psychiatry, as well as all inherited, obsolete psychological theories that may unduly influence the psychiatrist” (p. 121).

The latter two conditions, (c) and (d), of Baehr’s account of open-mindedness – namely that able and willing transcendence takes place “(c) in order to take up or take seriously the merits of (d) a distinct cognitive standpoint” (2011b, p. 212) – also bears close conceptual links with phenomenology. The third condition, (c), of Baehr’s account is also captured by other proponents of open-mindedness. For example, Hare (2011, 2003) suggests that the notion of “critical receptiveness” (which he borrows from Bertrand Russell) that open-mindedness captures, is “a readiness to consider new ideas together with a commitment to accept only those that pass scrutiny” (Hare, 2003, p. 79). Taking seriously the merits of alternative standpoints and the consideration of new ideas or perspectives needs to be guided by one’s “best judgement”. Open-mindedness in this regard therefore requires an attempt of reconciling “the tension between the conflicting demands of receptiveness towards new ideas and a critical assessment of those ideas” (Hare, 2009b, p. 38).

From the phenomenological perspective this notion of critical receptiveness brings to the fore Jaspers’ emphasis on “subjective analysis”. Indeed, the clinician *qua* seeker is called not to blindly accept the personal accounts provided by the patient *qua* knower, but to be an “investigator of meaning”. Thus, the clinician is required to engage in analysis – or critical receptivity or scrutiny – with the direct descriptions provided, in order to gain insight to, and understanding of, the nature of the psychic experience itself (Jaspers, 1968; Upthegrove et al., 2016, p. 88). The phenomenological process necessarily requires “scrutiny of the diverse and varied nature of what is really there in the patient’s experience” (Stanghellini & Broome, 2014, p. 170).

Thus, for phenomenological psychopathology, Baehr’s (d) condition (the “distinctive cognitive standpoint”) is the patient *qua* knower’s “subjective experience” and description or presentation of the “concepts and ideas which form the inner representation of psychic processes” (Jaspers, 1968, p. 1314). As Hare writes, “since open-minded inquiry requires consideration of the best evidence available, the fact of epistemic dependence inevitably means that we must turn to experts for a great deal of our knowledge” (2009b, p. 39). In the case of phenomenological psychopathology, this requires a repositioning of power, authority, and expertise. The patient is no longer viewed as a “person with a mental illness” receiving treatment from a psychiatric expert. Rather, they are repositioned, and seen as patient *qua* knower, expert of their subjective experiences. Similarly, the clinician is repositioned as the seeker of truth, knowledge, and understanding regarding the lived and subjective experiences of psychic phenomena. The clinician thus necessarily engages in processes of critical receptivity or scrutiny *because* the very nature of their investigation requires engaging with novel information, which is retrieved from a distinct cognitive standpoint (namely experience of the patient *qua* knower).

Importantly, the practices that are associated with open-mindedness (e.g., to consider new evidence, and to set aside theoretical constructs and one’s own preconceptions) are undertaken because they “greatly enhance our prospects of making progress in the direction of truth” (Hare, 2009b, p. 38). As such, the outcomes of the associated practices – or indeed the entire open-mindedness enterprise – are the very same as those that nourish our motivation to employ them to begin with: “a sincere commitment to the pursuit of the truth” (Hare, 2009b, p. 38).

As a virtue, open-mindedness exists as the “golden mean” between two vices. On one end of the spectrum, exist the intellectual vices of bias, prejudice, dogmatism, closed-mindedness, or narrow-mindedness. On the other end of the spectrum exist the intellectual vices of gullibility, credulity, or empty-mindedness (Baehr, 2011b; Hare, 2009b). At the former end of the spectrum, little to no novel information, evidence, or distinct cognitive standpoints are given merit or consideration. On the latter end of the spectrum, every possibility is considered worthy of investigation, whether fantastic or ridiculous, and serious inquiry becomes severely jeopardized. Indeed, open-mindedness is offered as an antidote to these intellectual vices, in the same way that Jaspers conceived of phenomenological psychopathology as an antidote to the over-reliance on objective symptoms that dominated psychiatry at the time, examined in often biased, prejudiced, and narrow-minded ways to fit predetermined diagnostic criteria.

## Training clinicians for open-minded phenomenological psychopathology

How then might open-mindedness be cultivated for phenomenological inquiry? In response, I focus on: (i) The psychiatric interview; and relatedly, (ii) three educational recommendations for phenomenological interviewing: cultivating the skills of good questioning, listening, and reflecting^22^^,^^23^.

The psychiatric interview, which the psychiatric medical encounter centers around, is the origin of the therapeutic relationship: it is where dynamic processes of understanding (clinical assessment or “diagnosis”) and therapy (“treatment”) commence. For phenomenological psychopathology, this dialogical process takes the form of a “relationship”, an inquiry that relies upon the patient *qua* knower to share their subjective, psychic experience, and the clinician’s skill of eliciting this information (Gadamer, 1996). The patient provides the rich, lived experiences of their life world, and the clinician subjects what is provided to analysis (such as questioning, contextualizing, interpreting, and challenging) in order to enrich their shared understanding (Jaspers, 1997; Spencer & Broome, 2023). This relationship and dialogical exchange rely heavily, I suggest, upon three core skills: questioning, listening, and reflecting.

I take the psychiatric interview^24^ and these three associated skills as the springboard for the educational recommendations I propose for the training of clinicians for open-minded phenomenology. My aim is to recommend that these skills are more intentionally cultivated in the education and training of clinicians for open-minded phenomenology. Due to limitations in scope, the particularities of *how* to educate for these skills are not discussed.

### The psychiatric interview

The medical encounter in psychiatry begins with a question, and questioning remains of central importance throughout. As Spencer and Broome (2023) explicate, the opening question – in what way are you feeling unwell? – can be explored and interpreted in two distinct ways. For somatic medicine, the question informs a diagnostic process that incorporates patient testimony and physical examination. Yet for psychiatry, the question takes on a unique quality, triggering “the start of an excavation into the internal and ineffable mental life of the psychiatric patient” (Spencer & Broome, 2023, p. 4). Our interest sits with this latter quality. The opening question, and those that follow – or, more crucially, the clinician’s *skill* of questioning – is core to both the psychiatric interview and the therapeutic relationship (Mundt, 2005; Stanghellini, 2007).

The often considered “gold standard” for the psychiatric interview has, both historically and contemporarily, been considered one that is highly structured, and which enables clinical diagnosis (Nordgaard et al., 2013). The focus of the interview (and the role of the questions that are asked) is on efficacy and reliability for the purpose of diagnosis. Indeed, as guided by mainstream diagnostic manuals (such as the DSM), a focus on symptom counting and checklists have had a fundamental influence on the clinical interview, and altered the ways in which clinicians view their patients (Broome, 2008). Yet this view of the psychiatric interview is increasingly being critiqued.

Stanghellini (2004) suggests that methodological concerns about the psychiatric interview mirror those of a traditional dichotomy within psychiatry and mental health. From the mainstream, technical perspective, the psychiatric interview is considered a *technique*, the intention of which is to elicit signs and symptoms that enable nosographical diagnosis: a method that is objective and reliable. From the alternative perspective, the interview is concerned with interpersonal *rapport* and the exploration of personal problems that arise within. What critics claim lacks in appraisal of both approaches is the overarching philosophical concern of ontology, epistemology, and ethics related to the psychiatric interview (Fuchs & Dalpane, 2022; Henriksen et al., 2022; Nordgaard et al., 2013; Stanghellini, 2004, 2013).

### Educating for good questioning

Indeed, there remains a lacuna in the literature concerning the psychiatric interview, with more interest in *what* should be assessed, rather than *how* the assessment should be conducted (Stanghellini, 2013). Stanghellini suggests two reasons for this neglect. Firstly, because “the skill in asking questions and listening to answers is taken for granted as a commonplace habit of everyday life” (Lazarsfeld, 1935; Stanghellini, 2013, p. 321). Secondly, because of “the assumption that interviewing is an art that cannot be taught but only acquired (MacKinnon and Michels, 1971; Stanghellini, 2013, p. 321). The concerns raised by Stanghellini above are echoed in Matthew Broome’s (2008) writing on Psychiatric interviewing:

Teaching clinical psychiatry to undergraduates and postgraduates can be a daunting process, both for the tutor^25^ and the students. For the medical student, the commonest reaction, after an outline of the psychiatric assessment is “How will I remember to ask all *that?*” (p.107)

In the transition from medical student to trainee psychiatrist, the consideration instead becomes “but which bits are *important*” (Broome, 2008, p.107). This change in reaction reveals, Broome suggests, a recognition that merely asking questions is not sufficient. Rather, an assessment requires a coherent narrative that has meaning for both patient and clinician, and careful attention to the selection of questions (also see Adler, 2004).

Questioning, quite clearly, is crucial to the psychiatric endeavor, and especially so for phenomenology. Lani Watson (2022) argues that questioning – particularly *good* questioning – is also fundamental to exercising open-mindedness. Furthermore, questioning may be viewed as the manifestation of open-mindedness, such that open-mindedness is evidenced by the asking of good questions.

As Stanghellini aptly touches upon, the skill of questioning is often taken for granted and assumed to be a commonplace, everyday habit. Similarly, Watson (2018) maintains that questioning is essential to our social, intellectual, and collective endeavors. The skill of questioning is clearly crucial to psychiatry and the phenomenological endeavor too. Yet, as Watson (2018) suggests, not all questions are equally effective, nor are all questioners equally adept. Good questioning requires proficiency and judgment – addressing not only *what* to ask, but also *how*, *when*, and *where*. Good questioning is, in short, an intellectual skill, and – contra concerns that it is an art acquired over time – one that can be educated (Watson, 2018, 2019, 2022).

The skill of questioning in this respect results not in an extensive list of questions that clinicians could ask (see Broome, 2008; Henriksen et al., 2022; Nordgaard et al., 2013). Rather, the educational endeavor is to help trainee clinicians understand the relationship between concepts in phenomenological psychopathology, intellectual virtues (such as open-mindedness), and the skill of questioning. In the case of the training and education of clinicians, educating for good questioning thus serves two important purposes for phenomenological psychopathology. Firstly, it contributes to the formation of intellectual character of the clinician in training, equipping them with a skill that enables excellence and success in their inquiry of subjective psychic phenomena. Secondly, it stimulates intellectually virtuous inquiry, namely open-mindedness (among other intellectual virtues), in the phenomenological endeavor.

### Educating for good listening

If one is to become competent at asking good questions in ways that enable successful open-minded phenomenological inquiry, it follows that there might equally be a skill in listening to the responses (Stanghellini, 2013). Indeed, it seems plausible that without good listening, the act of good questioning would likely fall short of open-mindedness and excellence in the phenomenological endeavor (Watson, 2022). Like questioning, listening may also be viewed as a commonplace, everyday activity. As Notess (2019) suggests, “listening as an active process is obscured by its comparative unobservability relative to the activity of speaking” (2019, p. 621). Its inconspicuousness makes listening easy to overlook: it is internally contained, less obvious (Notess, 2019). Yet, like questioning, it may be argued that listening is also a skillful action. It features throughout our lives and structures our treatment of others. Good listening is deeper than the perceptual task of hearing what the other says, it requires of the hearer to absorb and think seriously about the others’ words. It thus becomes a social task, establishing a relationship with the speaker that is open and responsive (Cohen, 2014; Notess, 2019).

For Stanghellini (2018), listening (and dialoguing) is crucial to the mediation of a therapeutically promising exchange. Indeed, the process of “unfolding” – “to open up and lay bare the pleats of the patient’s experiences” – relies upon listening and builds an ethic of “reciprocity and belonging” in the therapeutic relationship, as well as the generation of knowledge that stems from “subjective experiences and personal narratives” (Stanghellini, 2018, p. 960). Poor (inattentive) listening, on the other hand, serves to impede the affected individuals’ narrative, limiting them speaking “in their voice” and sharing the meanings of their experiences (Fusar-Poli et al., 2022, p. 183). In dialogue with a psychotic individual, the psychotic hallucinations or delusions may present as one voice amongst others. Indeed, in open-dialogue therapeutic encounters, where the affected individual “speaks in their own voice”, listening is considered more important than the manner of interviewing (Seikkula, 2019).

Recognizing the importance of listening in phenomenology, Spencer and Broome (2023) propose advancing Miranda Fricker’s account of “virtuous listening”.^26^ Virtuous listening is a form of listening that is “pro-active and socially aware”, and if exercised effectively will enable the clinician to create an “inclusive hermeneutical microclimate” (Fricker, 2007, p. 171).^27^ The challenges for such an interaction are well noted by Fricker, including the circumstances of the dialogical exchange – time availability, for instance – and most especially how much or little is shared with the clinician. Importantly, unlike Jaspers’ empathic listening, virtuous listening does not presuppose the possibility of “living” the others experience. Rather, when the patient narrates their phenomenological experience, the clinician should engage in a form of reflective listening (Spencer & Broome, 2023). Thus, the patient is not viewed as a “passive bystander”, but an epistemic agent engaging in a shared practice of meaning-making: something that is both core to the practice of phenomenology and to the exercise of open-mindedness. Accordingly, meaning structures of the psychic experience are gathered collectively by the clinician and patient working together, a process which is enabled when the clinician exercises virtuous listening – listening that is reflective, pro-active, and socially-aware – in order to grasp the patient’s lived experience.

### Educating for good reflecting

The final educational recommendation for open-minded phenomenology, namely reflection, has already featured briefly. Fricker’s (2007) account of virtuous listening requires the ability for one to do so “reflectively”. This reflection takes at least two forms. First, it requires the clinician to reflect on their position as a seeker of knowledge, instead of as an expert knower. And second, to reflect on *what* is said: the personal psychic experiences as narrated by the patient. For phenomenological psychopathology, “the form” of what is said – namely *how* it is said, or “the mode in which content is given to consciousness” – rather than the content itself, is often considered more important (Stanghellini & Broome, 2014, p. 170).

Reflection, like both questioning and listening, seems a ubiquitous everyday action. Similarly, its commonplace position may lead to the assumption that we all know how to do it. Yet surely the same qualifier for reflection exists as that of questioning and listening: that not all reflection is equally effective, nor are all individual’s equally adept at reflection. I thus propose that the skill analogy afforded listening and questioning also be applied to reflection. Indeed, the value of reflection in educational contexts is already well known, especially within medicine and social work (Aronson, 2011; Ferguson, 2018). Accordingly, while an everyday understanding of reflection may inform learning and insight, this is not equivalent to the “high level analysis, questioning, and reframing” that skillful reflection in educational and practice settings affords (Aronson, 2011, p. 1). Rather, it “is a metacognitive process that occurs before, during and after situations” that enable “greater understanding of both the self and the situation” – or psychic experience (Sandars, 2009, p. 685).

Schon (1983) formulated this as (i) “reflection *in* action”, when professionals consider their present context and think about what they are doing while they are doing it; and (ii) “reflection *on* action”, which occurs afterward, and involves linking practice to knowledge. Skillful reflection would thus enable the clinician to become aware of their presuppositions and how they constrain the way the clinician perceives and understands the patient’s subjective experience (Aronson, 2011). Furthermore, reflection enables the clinician to reformulate their assumptions, and can inform both *how* they listen to their patient, and the questions that they subsequently ask.

## Considerations

It is, however, also important to note that an intellectual virtue account of phenomenology does present some important limitations and barriers. These include the space and context within which clinicians work, and general limitations to intellectual virtue application. A particular concern (which also contributes to epistemic injustice) concerns structural limitations in healthcare systems and institutions. Principle among these are “serious resource limitations and time pressures” (Hassall, 2024, p. 4), resulting in short consultations and preferential use of standardized protocols (Kidd & Carel, 2017). Indeed, healthcare systems often prioritize a provision based on efficiency, influenced by fiscal concerns (Kidd & Carel, 2017). Clinical settings also involve “conditions of uncertainty and risk”, requiring decision-making based on available information (Kotzee, 2018, p. 505). These limitations present challenges to the application of intellectual virtues by clinicians. Open-mindedness, expressed through skilled questioning, listening, and reflecting, for example, will likely require time and capacity that clinicians cannot afford. Furthermore, such constraints in the healthcare system will also influence clinicians’ educational environment, preparing clinicians for work in the system as it exists, rather than its ideal form. Such constraints may also exist in the context of clinician education and training, rendering these recommendations “overly demanding” (cf. Porter, 2016). Indeed, a virtue ethical stance on healthcare provision, that is based on “right action” – which “comes from good or virtuous motivation involving benevolence or caring” (Slote, 2001, as cited in; Van Zyl, 2013, p. 181) and directed toward human wellbeing (*eudaimonia*) – may exist more in the realm of ideal. However, this does not mean that the education or training of clinicians should be limited to the same extent that our healthcare provision is.

## Conclusion

Alongside efforts to renew phenomenological psychopathology for the 21st century, this paper seeks to advance an account that supports successful investigation and inquiry into lived psychic experience. Counter to Jaspers (1968) claim that subjective experience can be accessed by empathic understanding alone, I advocate for an intellectual virtue account of phenomenological psychopathology as a means of ensuring excellence in the pursuit of its epistemic goals. Such an account necessarily involves intellectual effort and cognitive skill. Open-mindedness is used as an example to illustrate the value of incorporating intellectual virtues into phenomenological discourse. Such incorporation bolsters the epistemic agency of the “patient” as expert of their experience and the role of the clinician *qua* seeker: investigator of subjective experience. Success in phenomenological inquiry cannot be assumed. Rather, I suggest a focus on cultivating the intellectual skills of questioning, listening, and reflecting in the education and training of clinicians for phenomenological psychopathology.

## Notes

1. This framing intentionally seeks to avoid the use of terms such as abnormal, illness, and psychopathology due to its unhelpful nature and alignment with ableist and sanist perspectives. However, these terms do feature within this paper at times.

2. Stanghellini (2013) suggests that “praise of objectivity” and “avoidance of subjectivity” is not exclusive to psychiatric practice of the early twentieth century, and that an “objectifying trend” is also evident in clinical psychotherapeutic practice of the 21st century.

3. See Ratcliffe (2012) for an explication on the complexity of various conceptualizations of Husserlian accounts of empathy.

4. Importantly, while seeking to recognize patient *qua* knower and clinician *qua* seeker, and emphasizing these roles in this paper, this should not disregard that a clinician is of course a “knower” too. In this instance, to establish such a concrete dichotomy is problematic for three reasons. First, a clinician is a “knower” of what Jaspers would characterize as “objective knowledge” regarding mental ill health (in contrast to the subjective knowledge of a lived experience). Second, some clinicians may also have lived experience of the mental ill health that they are examining, in which case they would also be a “knower” of “subjective knowledge”. Third, the patient is as much part of the hermeneutic endeavor as the clinician – they too are seeking meaning. In this regard, good hermeneutical inquiry in the clinical encounter often occurs in tandem, with clinician and patient engaging in process of co-production of meaning making.

5. Related to the above footnote, while the patient is indeed a “knower” of their experience, knowledge – and articulation – of the experience may still be elusive to them. If this is the case, they too are seeking understanding. Indeed, the subjective experience of psychological phenomena can often be obscure, even to the patient (see e.g., Spencer, 2023).

6. Importantly, Ratcliffe (2012) reminds us that many first-person accounts of subjective experience, for example depression, are indescribable, and sometimes cannot be shared, exacerbating feelings of alienation and estrangement.

7. For Ritunnano (2022) phenomenology requires an openness and acceptance of possible competing views to co-exist “in the absence of any ultimate truth … [and the ability to] entertain the possibility that what someone is saying may not accurately reflect the ‘objective’ status of some external events … but may carry informational value which is of significance for a particular individual in the context of their own life narrative and circumstances” (p.257).

8. In discussion of virtue, Aristotle referred to these as dispositions of excellence that relate to one’s character (2002, p. 110); however, the term “virtue” is typically used in contemporary virtue ethics and moral psychology.

9. Julia Annas (2011) and Heather Battaly (2015) offer detailed explanations on virtue cultivation, which I recommend for novices to virtue and related ethical theories.

10. See Kotzee (2018) on virtue epistemology and clinical medical judgment.

11. Pritchard (2016) also offers helpful clarification on this matter.

12. All of which I suggest fall within the scope of knowledge acquisition that depends upon inquiry.

13. Which is not to suggest that subjective experiences be sought without concern for the social and political situatedness of the knower.

14. Importantly, this description of “vague and unconscious understanding” is not, to my reading of Jaspers (1968) work, intended to minimize its value, but rather to contrast it with the unachievable “science of psychology” in which “its ideal is a fully conscious understanding of mental processes … that can be presented in definite terms and forms” (p. 1315). Indeed, Jaspers argues, that “we must realize … that psychology cannot hope to approach this scientific ideal” (1968, p. 1315).

15. My emphasis in this paper is to address the intellectual virtues necessary (and perhaps inherent) to phenomenological psychopathology as it applies to the clinician *qua* seeker. That is not to say that such intellectual virtues are not necessary, or characteristic of the patient *qua* knower. Indeed, it seems likely that the dynamic relationship inherent to phenomenology would benefit from both parties exercising the intellectual virtues.

16. While I focus on testimonial injustice, it is important to note that testimonial injustice sustains *hermeneutical* injustice – when dominant societal discourses lack necessary vocabulary, resulting in one’s inability to articulate an experience or position. The latter also crucially needs addressing.

17. Exercising intellectual virtue thus supports the right recognition of the patient as expert of their subjective experience, as “*psychopathologia prima”*, and foundational to the generation of truth, knowledge and understanding (Stanghellini & Broome, 2014, p. 170). Phenomenological psychopathology is heralded as a champion of epistemic justice (Spencer & Broome, 2023). I hope it is evident in this paper that an intellectual virtue account of phenomenology, especially one of open-mindedness, also champions epistemic justice.

18. Baehr (2011a) suggests that intellectual integrity is the intellectual virtue most appropriate for this demand, which he views as supervening, or parasitic. However, my use of intellectual humility and open-mindedness correspond with accounts of these intellectual virtues by Baehr (2011b), Riggs (2010), and Spiegel (2012).

19. In his conceptualization of Dasein, Heidegger considered it a “basic openness to the world”, a “capacity to find the world meaningfully articulated” (Fernandez, 2019, p. 31) – important for psychology and psychiatry.

20. For other accounts of open-mindedness cf (Hare, 2009b, 2011; Kwong, 2016, 2017; Riggs, 2010).

21. Jack Kwong (2017) suggests that, for “openness”, or being “open”, to count as open-mindedness it needs to meet certain conditions. The mere similarity between the terms due to the inclusion of the word “open” should not allow for conflation between the terms: open-mindedness differs from merely being “open” in that it requires the agent to give serious consideration to opposing or novel perspectives (Kwong, 2017, p.1618).

22. *Should* such a response be elicited in clinicians’ professional education and training, this may initiate broader institutional reformation, enabling the type of structural, policy change required to address hermeneutical injustice (cf. Anderson, 2012; Christman, 2012). Indeed, institutional policies implemented in response to social injustice necessarily need to support individual virtues in local transactions (Christman, 2012).

23. Experience is considered a fundamental component of virtue development in adults, especially the meta-intellectual virtue of practical wisdom or phronesis (Darnell et al., 2019; Kotzee, 2018; Kristjánsson, 2015). As such, individual differences, including varied life experiences, age, culture, and background, will also contribute importantly to the development of an intellectually virtuous phenomenology. Such considerations might weigh into admissions decisions; however, my focus here is on educational recommendations for virtue development.

24. It is possible that the exercise of intellectual virtues more broadly might enable clinicians to be open-minded to consideration and application of novel and alternative therapeutic exchanges, such as the social psychiatry model associated with Frantz Fanon (Adams, 1970) and the sociogenesis of pathology (Zahavi & Loidolt, 2022).

25. Exemplarist thinking suggests that inculcating intellectual virtues requires role-modeling by exemplars, an educational recommendation strongly advocated for by virtue epistemologists (cf. Kotzee et al., 2021).

26. Elizabeth Anderson (2012) critiques Fricker’s suggestion for individual virtue as a response to hermeneutical and testimonial epistemic injustice, identifying the need for collective (i.e., structural and policy level) response to some epistemic harms, especially hermeneutical injustice.

27. Ritunnano (2022) argues convincingly “that the adoption of a critical phenomenological stance may improve conditions of hermeneutical marginalization as lived by individuals affected by psychiatric disorder” (p.243), which supports my proposition.
